# Post-hemorrhagic ventricular dilatation: Comparison of management pathways among North American level IV NICUs

**DOI:** 10.1038/s41372-026-02595-z

**Published:** 2026-02-23

**Authors:** Kristen Coletti, Stephanie S. Lee, Susan Cohen, Maria L. V. Dizon, David S. Hersh, Ulrike Mietzsch, Eylem Ocal, Elizabeth K. Sewell

**Affiliations:** 1 Department of Pediatrics, University of Pennsylvania Perelman School of Medicine, Division of Neonatology, Children’s Hospital of Philadelphia, Philadelphia, PA USA; 2 Department of Pediatrics, Division of Neonatology, University of Iowa Carver College of Medicine, Stead Family Children’s Hospital, Iowa City, IA USA; 3 Department of Pediatrics, Division of Neonatology, Medical College of Wisconsin, Children’s Wisconsin, Milwaukee, WI USA; 4 Department of Pediatrics, Northwestern University Feinberg School of Medicine, Division of Neonatology, Ann & Robert H. Lurie Children’s Hospital of Chicago, Chicago, IL USA; 5 Division of Neurosurgery, Connecticut Children’s, Hartford, CT USA; 6 Department of Neurosurgery, UConn School of Medicine, Farmington, CT USA; 7 Department of Pediatrics, Division of Neonatology, University of Washington School of Medicine, Seattle Children’s Hospital, Seattle, WA USA; 8 Department of Neurosurgery, Division of Pediatric Neurosurgery, University of Arkansas For Medical Sciences, Arkansas Children’s Hospital, Little Rock, AR USA; 9 Department of Pediatrics, Emory University School of Medicine and Children’s Healthcare of Atlanta, Atlanta, GA USA

**Keywords:** Paediatric neurological disorders, Hydrocephalus, Brain injuries, Neurological manifestations

## Abstract

**Objective:**

To assess the proportion of Level IV NICUs with post-hemorrhagic ventricular dilatation (PHVD) management pathways and compare the pathways.

**Study design:**

A survey was distributed to 49 Children’s Hospitals Neonatal Consortium (CHNC) Level IV NICUs. A summarized pathway was developed from written pathways.

**Result:**

Survey response rate was 82%. Twelve (30%) NICUs have written pathways, 11 (28%) report informal consensus, and 17 (43%) lack consensus. Among the 12 written pathways, all serially monitor ventricular dilatation on cranial ultrasound (CUS) using ventricular index (58%) or frontal-occipital-horn-ratio (33%). Threshold for surgery varies: 33% of sites rely on CUS alone, while 67% incorporate clinical symptoms. Half of sites use lumbar puncture to decrease PHVD before surgery. Criteria for converting temporizing to permanent shunt is present in 67% of pathways.

**Conclusion:**

Amongst centers with written PHVD pathways, variable monitoring and intervention criteria exist. Most NICUs lack formal pathways, demonstrating opportunities to standardize care.

## Introduction

Post-hemorrhagic ventricular dilatation (PHVD) is a severe complication of prematurity, resulting in neurodevelopmental impairment and death in up to 68% of affected neonates [1, 2]. Preterm infants with grade III or grade IV intraventricular hemorrhage (IVH) are at highest risk for progressive PHVD [3], which may lead to increased intracranial pressure and subsequent neurological injury. Surveillance imaging with cranial ultrasound (CUS) is a mainstay of neonatal neurocritical care to identify patients who may benefit from intervention [4–6].

The definitive treatment for progressive PHVD is cerebrospinal fluid (CSF) diversion, typically via a ventricular shunt. However, many preterm infants with PHVD have concomitant issues that preclude shunt placement, including necrotizing enterocolitis, extensive IVH, and low relative weight at presentation [7]. Temporizing interventions are typically recommended for these patients and may include a ventricular access device (VAD), a ventriculosubgaleal shunt (VSGS), or neuroendoscopic lavage [4, 7, 8]. Despite these interventions, most infants with progressive PHVD go on to need permanent CSF diversion [9, 10].

Interventions and their timing are selected based on clinical judgment, prior experience, and personal or institutional preference, with practice variability within and across different centers [4, 9, 11–15]. Published guidelines and management protocols for PHVD exist [4, 8, 9], though it is unknown whether and how different institutions adopt them. In 2021, our group surveyed 20 North American level IV NICUs within the Children’s Hospitals Neonatal Consortium (CHNC) [12]. Survey respondents from most centers reported the presence of institutional protocols to manage PHVD. Yet, differences in surveillance, treatment and follow up were identified between institutions, as well as between neonatologist and neurosurgeon pairs from the same institution. It is unknown whether this management variability reflected deviations from institutional protocols, a lack of protocolized consensus regarding certain aspects of care, or differences in the institutional protocols themselves. Hence, the objectives of this study are to: (1) assess the proportion of NICUs with formal, written clinical pathways for the management of PHVD, and (2) identify areas of agreement and heterogeneity between the written pathways by consolidating the individual pathways into a single summarized pathway.

## Methods

The CHNC is a nonprofit organization comprised of 49 North American Level IV NICUs dedicated to improving outcomes for medically complex neonates and infants through data sharing, benchmarking, and research. There are approximately 152 Level IV NICUs in the United States [16], thus CHNC represents ~1/3 of Level IV NICUs in North America. Greater than 95% of CHNC sites have pediatric neurosurgeons, radiologists and sonographers. Members of the CHNC Neurosurgery Focus Group, which includes neonatologists, neurosurgeons, nurse practitioners, and data abstractionists, designed a cross-sectional survey consisting of 12 questions about center demographics, PHVD pathway development, and pathway utilization. Centers with written PHVD pathways submitted their pathways for analysis. The Institutional Review Board at Children’s Healthcare of Atlanta approved this study (STUDY00002223).

The survey was administered via Qualtrics and was distributed by e-mail to a representative neonatologist from each of the 49 CHNC sites. Neonatologists from either the CHNC Neurosurgery Focus Group or from the list of CHNC site sponsors were contacted. While the identified neonatologist could forward the survey to another physician for completion, a maximum of one survey response per site was included. The survey remained open from June to October 2024, and reminder emails were sent at regular intervals.

Survey responses were analyzed using descriptive statistics and were summarized as percentages of respondents. Continuous variables were presented as medians with ranges (Microsoft Excel, 2025). Pathways were compared to identify similarities and differences regarding eligibility criteria, monitoring of PHVD, timing and type of intervention for PHVD, and follow-up. A summarized pathway was developed, presenting the aggregate count and percentage of written pathways addressing each theme.

## Results

Physicians from 40 of the 49 CHNC centers completed the survey resulting in an 82% response rate. Institutional and pathway characteristics are presented in Table 1. Of the 40 responding centers, 12 (30%) reported having written PHVD pathways, 11 (28%) reported having informal consensus regarding management strategies, and 17 (43%) lacked consensus. Pathways were implemented between 2015 and 2023, with the median year being 2020. Among the 12 sites with written pathways, 7 (58%) care for outborn infants only, and 5 (42%) care for both inborn and outborn infants. All 12 sites reported that their pathways are written and used by neonatologists and neurosurgeons.

**Table 1:** Institutional and Pathway Characteristics.

Variable	*N* (%) or Median (range)
**Institutional** (***n*** = **40**)	
Institutional region	
Midwest	13 (32.5%)
Northeast	6 (15.0%)
Southeast	11 (27.5%)
Southwest	4 (10.0%)
West	6 (15.0%)
Unit size	
Less than 41 beds	9 (22.5%)
41 to 60 beds	14 (35.0%)
61 to 80 beds	3 (7.50%)
81 to 100 beds	8 (20.0%)
More than 100 beds	6 (15.0%)
Admission type^a^	
Inborn only	1 (2.6%)
Outborn only	19 (50.0%)
Inborn and outborn	18 (47.4%)
Pathway implemented	
Yes, written	12 (30%)
Yes, consensus, not written	11 (27.5%)
No	17 (42.5%)
**Pathway Characteristics** (***n*** = **12**)	
Year of implementation^b^	2020 (2015–2023)
Year of most recent revision	2022 (2020–2023)
Written by:^b^	
Neonatologists only	0
Neurosurgeons only	0
Both	11 (100%)
Used by:	
Neonatologists only	0
Neurosurgeons only	0
Both	12 (100%)
Perceived inconsistencies in pathway ^b^	
Yes	3 (27.3%)
No	8 (72.7%)

^a^Missing two centers (*N* = 38).

^b^Missing one center (*N* = 11).

### Summarized pathway

#### IVH and PHVD surveillance

**Fig. 1:**
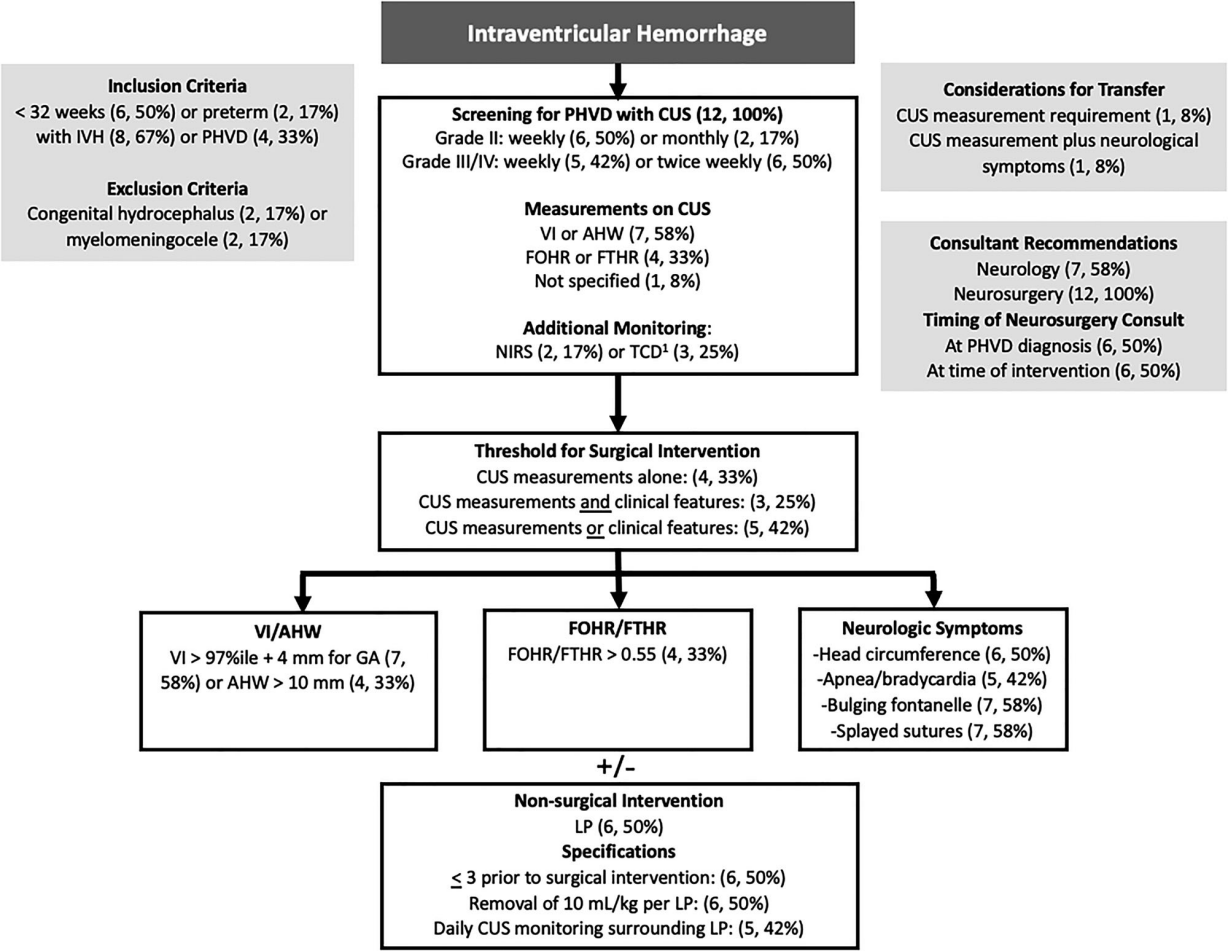
PVHD Screening, Non-surgical Management, and Intervention Thresholds. Pathway summarizing PHVD screening recommendations (*n*, %), *N* = 12. ^1^Explicitly mentioned in pathways separate from routine CUS monitoring; used to report resistive index which can correlate with intracranial pressure. CUS cranial ultrasound, PHVD post-hemorrhagic ventricular dilatation, VI ventricular index, AHW anterior horn width, FOHR frontal-occipital horn ratio, FTHR frontal-temporal horn ratio, NIRS near infrared spectroscopy, TCD transcranial doppler, GA gestational age, LP lumbar puncture.

Among the 12 centers with written pathways, all use CUS to screen for PHVD following IVH. However, the IVH grade and frequency dictating serial screening varies by center (Fig. 1). Most sites (92%) quantify the patient’s ventricular dilatation on CUS, with ventricular index (VI) being more commonly measured than frontal-occipital horn ratio (FOHR) and frontal-temporal horn ratio (FTHR) (58% versus 33%, respectively). CUS measurements impact neurosurgical decision-making at most centers, and there is consistency regarding which CUS measurements prompt surgical intervention: VI > 97%ile + 4 mm for gestational age or AHW > 10 mm, and FOHR/FTHR > 0.55 are uniformly chosen thresholds. However, the influence of clinical signs and symptoms of elevated intracranial pressure on surgical decision-making is variable: 33% of sites rely on CUS measurements alone, 42% on CUS measurements *or* clinical features, and 25% on CUS measurements *and* clinical features. Half of pathways recommend neurosurgery consultation at the time of intervention, and half at the time of PHVD diagnosis. Neurology consultation is recommended in 58% of pathways.

#### Intervention for PHVD

**Fig. 2:**
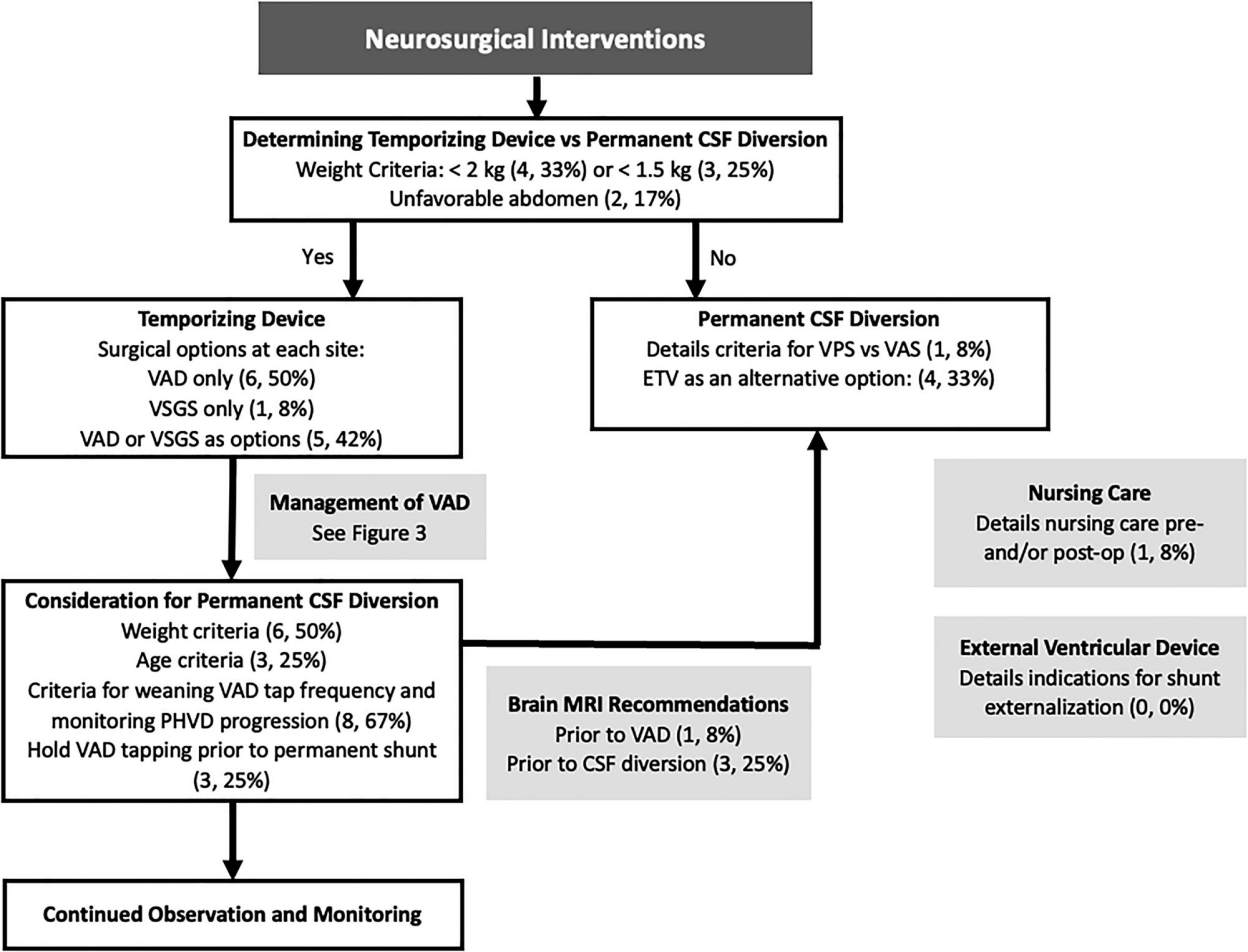
Neurosurgical Management of PHVD. Pathway summarizing neurosurgical temporizing and permanent cerebrospinal fluid diversion interventions, *N* = 12. Data presented as (*n*, %). VAD ventricular access device, VSGS ventriculosubgaleal shunt, VPS ventriculoperitoneal shunt, VAS ventriculoatrial shunt, ETV endoscopic third ventriculostomy, CSF cerebrospinal fluid, PHVD post-hemorrhagic ventricular dilatation.

Half of sites utilize lumbar puncture (LP) prior to neurosurgical intervention, with attempted removal of 10 mL/kg of CSF up to three times (Fig. 1). The initial neurosurgical intervention for PHVD may be a temporizing device or permanent CSF diversion (Fig. 2). The decision for one versus the other is most commonly determined by an infant’s weight. When a temporizing device is recommended, half of sites place VADs only, 8% place VSGS only, and 42% place either VAD or VSGS.

#### VAD management

**Fig. 3:**
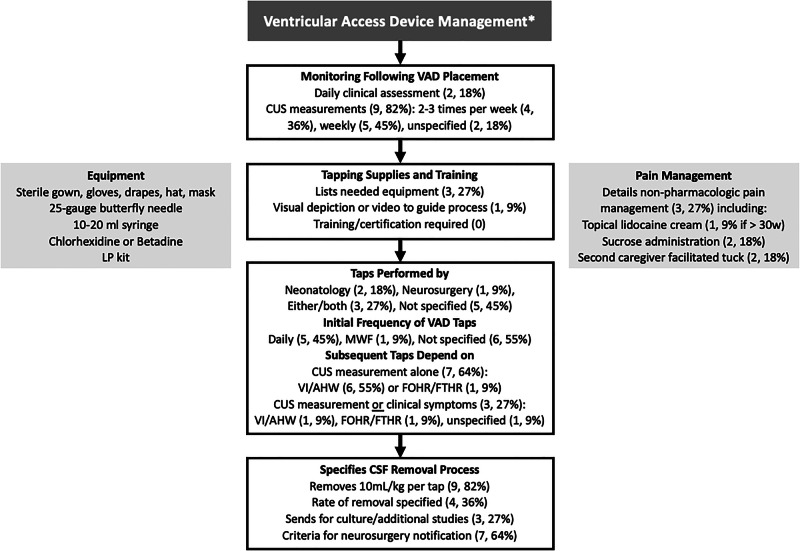
Management of VAD. Pathway algorithm summarizing VAD management, *N = 11, 1 site excluded from figure due to placement of VSGS only. Data presented as (*n*, %). VAD ventricular access device, CUS cranial ultrasound, LP lumbar puncture, MWF Monday Wednesday Friday, VI ventricular index, AHW anterior horn width, FOHR frontal-occipital horn ratio, FTHR frontal-temporal horn ratio.

Among sites that utilize VADs (*n* = 11), all describe VAD management after placement (Fig. 3). Most pathways (82%) include serial CUS after VAD placement. The frequency of serial monitoring varies from 1–3 times per week. Half of pathways recommend an initial frequency for VAD tapping, with daily being most common. Subsequent tapping intervals depend on CUS measurements alone (VI/AHW > FOHR/FTHR) in 64% of centers. However, 27% of centers specify that the frequency of tapping depends on either CUS measurements or clinical signs/symptoms. When specified, the tap volume is 10 mL/kg. The rate of CSF aspiration is not consistently specified. Equipment, training protocols, and peri-procedural pain management are rarely detailed.

#### Permanent CSF diversion

Many pathways specify age and/or weight criteria for converting a temporizing device to a permanent shunt (Fig. 2). Most centers assess the persistence of PHVD before permanent CSF diversion either through protocolized weaning of VAD taps (67%) or a mandated tapping holiday (25%). When permanent CSF diversion is indicated, few pathways (25%) specify an MRI recommendation before surgery. Whether to place a ventriculoperitoneal shunt, ventriculoatrial shunt, or perform an endoscopic third ventriculostomy (ETV) is infrequently specified.

#### Post-surgical evaluation

**Fig. 4:**
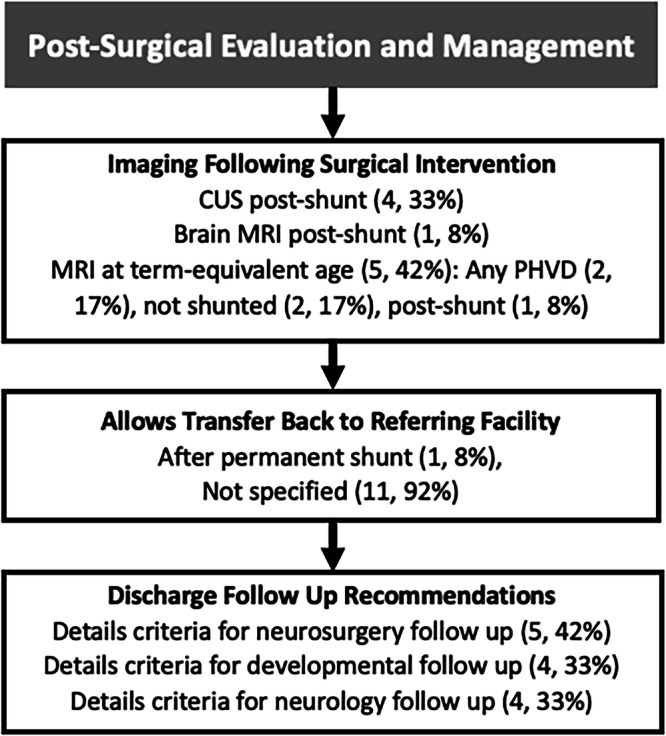
Post-Permanent CSF Diversion Management and Discharge Preparation. Pathway summarizing management after placement of permanent shunt. *N* = 12. Data presented as (*n*, %). VPS ventriculoperitoneal shunt, VAS ventricular atrial shunt, CUS cranial ultrasound, MRI magnetic resonance imaging, PHVD post-hemorrhagic ventricular dilatation.

Details regarding postoperative evaluations and discharge planning following permanent CSF diversion are uncommonly included in the pathways (Fig. 4). Less than half of the centers specify postoperative neuroimaging recommendations or follow-up criteria. The patients for whom a postoperative MRI is recommended, and the timing of this imaging, vary across pathways.

## Discussion

Only 30% of the 40 Level IV NICUs in North America that responded to this survey have written pathways for managing PHVD. Among these pathways, similarities and differences were identified. All pathways rely on serial CUS ventricular measurements to guide the need for and timing of interventions, including LPs, temporizing device placement, VAD taps, and permanent CSF diversion. There is consensus about using ventricular measurement thresholds to trigger intervention and using weight criteria to determine the need for an initial temporizing device versus permanent shunt. However, variability exists in CUS monitoring frequency, the specific ventricular measurement used, the role of clinical symptoms in neurosurgical decision-making, and post-intervention management.

While severe IVH is one of the few predictors of subsequent PHVD [1, 3, 17], the trajectory of progressive PHVD is variable, with just over half of infants requiring intervention [17, 18]. Thus, serial CUS monitoring is the mainstay of PHVD screening, as reflected in all institutional pathways analyzed in this study. However, we discovered a lack of consensus about how ventricular size is measured (VI/AHW versus FOHR/FTHR) and whether clinical signs and symptoms influence intervention timing. This variability may stem from differences between two widely disseminated PHVD studies published within the last decade. In 2017, the Hydrocephalus Clinical Research Network’s Shunting Outcomes in Posthemorrhagic Hydrocephalus (SOPHH) prospective cohort study utilized a standardized decision rubric whereby the decision to treat PHVD relied on a FOHR ≥ 0.55 *and* at least 2 of 3 symptoms (bradycardia, split sutures, and/or a bulging fontanelle). The rubric was followed with high compliance by neurosurgeons [7]. In our study, 33% of sites follow FOHR, and 25% of pathways recommend intervention based on symptoms alongside CUS findings, often following the SOPHH protocol. However, concerns exist that clinical symptoms of increased intracranial pressure develop after ventricular dilatation due to preterm infants’ high brain compliance and large extracerebral space [9, 19]. As such, in 2020, the Early vs. Late Ventricular Intervention Study (*ELVIS*) trial randomized patients to early or late intervention based on ventricular measurements alone. Earlier intervention at a VI above the 97%ile led to better neurodevelopmental outcomes than waiting for the VI to reach 97%ile + 4 mm, suggesting a benefit of intervention based on ventricular size *regardless* of clinical symptoms [2, 10, 20–22]. In our study, 58% of sites follow VI, and 33% of pathways recommend intervention based on CUS findings alone. These different management approaches in fundamental PHVD studies, may contribute to a lack of consensus between centers about CUS measures of ventriculomegaly and intervention thresholds, and may also explain why most centers lack formal, standardized approaches to PHVD care.

Given that evidence increasingly supports earlier intervention for PHVD [2, 20–22], a protocol proposed in 2020 by an international group of neurosurgeons and neonatologists prioritized early mitigation of ventricular dilatation on neuroimaging using LPs or surgery [9]. Our results demonstrate that this approach has not been universally adopted in North America. This may be because immense interdisciplinary collaboration is required to care for infants with PHVD making it logistically complex to adopt an early intervention protocol. Outborn infants must transport to referral centers, then be evaluated and scheduled for surgery while comorbid conditions are concurrently managed. A recent study aiming to maintain VI < 97^th^%ile + 4 mm using LPs then VADs, demonstrated that by the time of VAD placement, most infants were beyond the goal VI threshold (median 97^th^%ile + 13 mm) [18], exemplifying these complexities. Additionally, point-of-care ultrasound is not standard of care in North America, and frequent CUS monitoring 2-3 times per week [9, 20], and even daily in some protocols [18], is time and cost-intensive. At present, 42% of pathways recommend CUS measurements only weekly prior to surgery, and 45% weekly after VAD placement. Thus, frequent CUS monitoring may be an additional barrier. Alternatively, it is possible that benefits of early intervention are not universally accepted. This could be due to [1] the older age (median 27.4 weeks) and larger birth weights (median ~1.1 kg) of infants included in the *ELVIS* trial, limiting generalizability to the smallest, sickest infants, or [2] the non-significant difference in the primary outcome of VPS or death between intervention and control groups. Furthermore, with earlier intervention, more infants are potentially exposed to surgical complications, which may be an additional concern.

Alternatively, management variability identified in our study may simply represent the expected lag between the publication of new findings, and their incorporation into guidelines and institutional pathways. Some institutions may still rely on guidelines published by the Congress of Neurologic Surgeons (CNS) in 2014 and updated in 2020 [4, 8], particularly for scenarios where evidence is mixed. For example, the CNS guidelines recommended against serial LPs to prevent hydrocephalus progression [4], supported by a Cochrane review demonstrating no association between serial LPs and death or permanent shunt for infants with PHVD [23]. In contrast, the *ELVIS* trial and El dib et al. (2020) consensus recommendations incorporated LPs into management protocols as the first step to temporize ventricular dilatation progression [9, 20]. In our study, half of pathways recommend early LPs. Interestingly, post-discharge follow-up recommendations are infrequently specified in pathways, suggesting variable longitudinal surveillance in different locations and over time. Given the high rate of neurodevelopmental impairment in infants with PHVD, standardizing follow up is prudent.

This study has several limitations. Despite the high response rate (82%), only 30% of the responding institutions have written PVHD pathways. This restricted our analysis of PHVD management algorithms to 12 level IV NICUs across North America, which may limit the generalizability of our findings. Additionally, the institutional pathways analyzed in this study are not necessarily evidence-based clinical guidelines, but rather algorithms designed to standardize care within a single institution. These pathways are likely intended to be flexible so that clinical circumstances and the expertise of managing clinicians can influence care if evidence is scarce. As such, some pathway ambiguity is likely intentional. Last, we did not evaluate pathway adherence or clinician perception of pathways but focused solely on the written pathways themselves. Future work assessing the relationship between pathways, adherence, and outcomes may further delineate optimal care for PHVD.

## Conclusions

Institutional pathways standardize clinical care and improve clinical efficacy [24], and neonatologists and neurosurgeons have expressed willingness to adopt consensus-based guidelines [12]. Yet only 30% of Level IV NICUs in the CHNC have implemented standardized management pathways for PHVD. There is general agreement about using CUS for diagnosis and surgical decision-making. However, practice variation in ventriculomegaly measurements and intervention thresholds highlight ongoing opportunities for consensus. The lack of written management guidelines in 70% of centers highlights an opportunity for most centers to standardize care.

## Data Availability

The institutional pathways and datasets generated during and/or analyzed during the current study are available from the corresponding author on reasonable request.
